# Circulating Biomarkers in Medullary Thyroid Carcinoma: Bridging Laboratory Complexities and Clinical Application Through Algorithm Design

**DOI:** 10.3390/jcm14165645

**Published:** 2025-08-09

**Authors:** Luca Giovanella, Federica D’Aurizio, Petra Petranović Ovčariček

**Affiliations:** 1 Department of Nuclear Medicine and Thyroid Diseases, Gruppo Ospedaliero Moncucco, 6900 Lugano, Switzerland; 2 Department of Nuclear Medicine and Thyroid Centre, University Hospital of Zurich, 8091 Zurich, Switzerland; 3 Department of Clinical Chemistry and Endocrinology, Medysin, 6000 Luzern, Switzerland; 4 Department of Laboratory Medicine, Udine University Hospital, 33100 Udine, Italy; federica.daurizio@asufc.sanita.fvg.it; 5 Department of Oncology and Nuclear Medicine, University Hospital Center Sestre Milosrdnice, 10000 Zagreb, Croatia; p.petranovic@gmail.com; 6 School of Medicine, University of Zagreb, 10000 Zagreb, Croatia

**Keywords:** medullary thyroid carcinoma, calcitonin, procalcitonin, carcinoembryonic antigen, biomarkers, doubling time, immunoassay, pro-gastrin-releasing peptide, diagnostic algorithms

## Abstract

Medullary thyroid carcinoma (MTC) is a rare (~2–5% of all thyroid cancers) neuroendocrine thyroid malignancy originating from parafollicular C-cells of the thyroid gland with variable biological behavior and potential for early metastasis. Diagnosis, staging, and surveillance are heavily reliant on circulating biomarkers. We aimed to provide a comprehensive overview of circulating biomarkers in the management of MTC and propose an integrated, evidence-based algorithm to guide clinical decision-making using both established and emerging biomarkers. This is a narrative review on the evolving landscape of biomarker-driven management in MTC with emphasis on analytical advancements, clinical applications, and the prognostic implications of individual and combined biomarkers. Calcitonin remains the cornerstone biomarker for MTC, and new generation immunoassays have addressed several pre-analytical and analytical challenges such as pre-analytical degradation, inter-assay variability, and biological confounders. Procalcitonin (ProCT) has emerged as a stable and less interference-prone alternative or adjunct to calcitonin, which is particularly useful in cases with indeterminate calcitonin levels. Carcinoembryonic antigen (CEA) remains a useful complementary biomarker often correlating with aggressive behavior, advanced disease, and distant metastases. Kinetic evaluation (doubling times) of calcitonin and CEA offers independent prognostic information values and those < 6 months are associated with poor survival, whereas those > 2 years suggest favorable outcomes. Newer biomarkers such as pro-gastrin-releasing peptide (ProGRP) and carbohydrate antigen 19-9 (CA19-9) show potential in monitoring advanced disease and response to therapy. Their role is still under investigation but appears promising, particularly when used in conjunction with calcitonin and CEA. Our work advances a comprehensive and clinically pragmatic framework for the management of MTC by integrating established and emerging biomarkers with evidence-based algorithms, offering greater diagnostic precision, more reliable prognostic stratification, and improved personalization of follow-up and treatment strategies.

## 1. Introduction

Medullary thyroid carcinoma is a rare C-cell-derived neuroendocrine tumor accounting for ~0.5–1.5% of thyroid nodules, ~2–5% of thyroid cancers, and ~0.15% of thyroid nodules incidentally discovered during autopsies of subjects who died from thyroid-unrelated conditions [1]. MTC exhibits a strong genetic component, particularly through mutations in the rearranged during transfection (RET) proto-oncogene.

### 1.1. Hereditary MTC and Related Syndromes

Hereditary MTC forms (~25% of cases) are caused by inherited autosomal dominant germline pathogenic variants in the RET proto-oncogene and are subclassified into Multiple Endocrine Neoplasia (MEN) type 2A and type 2B and Familial MTC (FMTC), respectively. MEN2A includes MTC, pheochromocytoma, and hyperparathyroidism (due to parathyroid hyperplasia or multiple adenomas); MEN2B (more aggressive) combines MTC, pheochromocytoma, mucosal neuromas, and marfanoid habitus; and FMTC presents as isolated MTC without other endocrine tumors [2,3]. Hereditary MTC is typically multifocal and bilateral, with variable penetrance and biological/clinical behavior, respectively [4]. Finally, pediatric MTC is exceedingly rare, and inherited cases of MEN 2 are involved in almost all cases [5]. Notably, identification of different RET pathogenic variants improved understanding of the natural course of disease, allowing predictive diagnosis, prophylactic thyroidectomy in pathogenic variants carriers, and targeted therapy with selective RET inhibitors (i.e., selpercatinib and pralsetinib) in advanced or progressive RET-mutant MTC (Table 1).

### 1.2. Sporadic Medullary Thyroid Carcinoma

Sporadic MTC represents ~75% of MTC cases and it is usually unilateral and unifocal and presents later in life, often diagnosed due to thyroid nodules, cervical lymphadenopathy, and/or serum calcitonin screening. Notably, germline RET pathogenic variants (often “de novo”) are found in up to 5–7% of sporadic MTC cases that have major implications for familial inherited risk and management decisions. Moreover, about 50–60% of patients with sporadic MTC without germline pathogenic variants may carry somatic pathogenic variants, commonly involving codon M918T, which is associated with more aggressive disease and poorer prognosis. Finally, activating pathogenic variants in RAS genes (HRAS, KRAS, NRAS) have been identified in RET-negative sporadic MTC, often linked with a more indolent course compared to RET-driven tumors. Accordingly, genetic testing for RET pathogenic variants is recommended in all patients with a personal medical history of primary C-cell hyperplasia, MTC, or MEN 2 syndrome, respectively.

### 1.3. Clinical Presentation and Course of the Disease

MTC typically presents as a thyroid nodule, which is occasionally discovered by patients or physicians during a routine clinical examination. Currently, thyroid nodules are mainly detected during thyroid-unrelated imaging procedures, such as ultrasound (US) and vascular studies, computed tomography (CT), magnetic resonance (MR), and positron emission tomography (PET), coupled with CT (PET/CT) or MR (PET/MR). Diagnostic algorithms for MTC are still debated (see below). MTC can metastasize via both lymphatic and hematogenous routes, which contribute to a clinical course that is generally more aggressive than that of differentiated thyroid carcinomas and typically less severe than anaplastic thyroid carcinoma [6]. Treatment of MTC primarily involves total thyroidectomy with central neck lymph node dissection, as surgery remains the only curative option. More extensive lymphadenectomy may be required in cases of locally advanced or metastatic disease. Unlike differentiated thyroid cancers, MTC does not respond to radioactive iodine or thyroid-stimulating hormone suppression therapy. For patients with progressive or symptomatic metastatic disease, systemic therapies such as tyrosine kinase inhibitors (e.g., vandetanib and cabozantinib) have demonstrated clinical benefit by targeting RET pathogenic variants and other pathways involved in tumor growth. Recently, highly selective RET inhibitors like selpercatinib and pralsetinib have shown promising efficacy in RET-mutated MTC. Supportive care and close biochemical and imaging monitoring are essential for ongoing management and early detection of recurrence. Disease-specific mortality at 10 years has been reported to range between 13% and 40%, with overall 10-year survival potentially declining to as low as 50% in some instances [7]. Several prognostic factors, as older age and advanced tumor stage at presentation, have been associated with increased disease-specific mortality [8]. Conversely, early detection and the absence of extra-thyroidal extension are generally associated with more favorable outcomes, with survival rates reaching up to 90% over a 35-year period, as reported in some series [9,10]. Thyroid parafollicular C-cells produce calcitonin, a highly sensitive circulating biomarker adopted as standard of care for the diagnosis and the follow-up of MTC. Moreover, parafollicular C-cells also produce the calcitonin precursor ProCT and several peptides, such as CEA, ProGRP, chromogranin A, neuron-specific enolase, and CA19-9, respectively. Despite its pivotal role, calcitonin measurement is subject to several analytical limitations and interferences that may lead to false-positive or false-negative results, complicating the interpretation of calcitonin results and potentially impacting clinical decisions [11]. Some alternative biomarkers have been proposed to complement or even substitute calcitonin in evaluating MTC patients. This is a narrative review on the evolving landscape of biomarker-driven management in MTC, with emphasis on analytical advancements, clinical applications, and the prognostic implications of individual and combined biomarkers. We aimed to provide a comprehensive overview of circulating biomarkers in the management of MTC and advance a comprehensive and clinically pragmatic framework for the management of MTC by integrating established and emerging biomarkers with evidence-based algorithms to offer greater diagnostic precision, more reliable prognostic stratification, and improved personalization of follow-up and treatment strategies.

## 2. Biomarkers of Medullary Thyroid Carcinoma

### 2.1. Calcitonin

Parafollicular thyroid C-cells uniquely secrete calcitonin, a 32-amino-acid peptide hormone, making serum calcitonin levels pivotal for the management of MTC. Serum calcitonin assays include radioactive (IRMA) and non-radioactive (IMA) immunometric methodologies, each varying in sensitivity, specificity, and susceptibility to interferences. Further complicating quantification, calcitonin circulates as multiple isoforms and fragments that some assays fail to detect, impairing inter-assay comparability. Notably, new-generation calcitonin assays, particularly the latest immunochemiluminometric assays (ICMAs) on automated platforms, have significantly enhanced analytical performance, thereby improving their clinical utility. In particular they minimize interferences with calcitonin precursors, enhancing specificity for mature monomeric calcitonin. Moreover, they enable ultrasensitive detection (i.e., <2 pg/mL), allowing for the reliable identification of subtly elevated basal calcitonin levels [11]. Finally, automated platforms offer high throughput, high sensitivity, and full automation, often integrating with laboratory automation systems. They streamline the testing process, reducing manual intervention and potential errors [12]. Additionally, calcitonin measurement also presents analytical challenges which can reduce assay accuracy and inter-method comparability [13], as detailed here.

- *Pre-analytical factors:* Serum proteases can degrade the calcitonin molecule, necessitating rapid sample processing, refrigerated storage, or use of protease inhibitors [14].
- *Biological confounders:* Hypergastrinemia, chronic renal insufficiency, proton-pump inhibitor therapy, cigarette smoke, pregnancy, and lactation may elevate calcitonin in the absence of MTC [15].

Moreover, mild calcitonin elevations can be observed in non-malignant conditions, including C-cell hyperplasia, various non-thyroid neuroendocrine neoplasms (i.e., pancreatic NETs), leukemias, systemic mastocytosis, small-cell lung carcinoma, breast and pancreatic cancers, renal dysfunction, primary hyperparathyroidism, and autoimmune thyroiditis (even if the latter association is currently debated) [16,17].

- *Inter-assay variability:* Comparability across platforms remains imperfect; consistent laboratory/method use is critical for serial monitoring [18].
- *Immunoassay interferences:* Rare heterophilic antibodies may still skew results, underscoring the importance of selecting the appropriate assay and performing repeat testing. Serum pretreatment in heterophilic antibody blocking tubes may detect the interference by lowering the measured calcitonin [19].
- *Hook effect:* It may paradoxically under-report calcitonin in patients with very high levels; its mitigation requires serial serum dilution protocols in high-range samples [20].

#### 2.1.1. Calcitonin Stimulation Testing

In order to increase the specificity of serum calcitonin values and decrease false-positive results, calcitonin stimulation testing was proposed and widely adopted in the past. Traditionally, stimulation with pentagastrin was used with the intention to exclude MTC in patients with basal calcitonin in the gray zone (i.e., 10–100 pg/mL) and detect a residual response after surgery which indicates the need for additional, more aggressive, reinterventions. Pentagastrin has not been available for many years and high-dose calcium gluconate test (2.5 mg/kg) has been proposed as a reliable alternative. It is an effective stimulus which is even more potent than pentagastrin with fewer side effects. Notably, setting reliable cutoffs for either pentagastrin or calcium-stimulated cutoff values was substantially unsuccessful, with large differences reported in the literature. Recent studies have proven that stimulating calcitonin offers, in general, no additional value compared to basal-CT measurement with new generation immunoassay. On the other hand, it may be useful in selected cases, especially in determining the timing of prophylactic thyroidectomy in carriers of germline pathogenic RET variants [21].

In summary, the new generation calcitonin assays improve early detection, reduce false-positives, and simplify the diagnostic trajectory of MTC. Greater baseline sensitivity and gender-specific thresholds further narrow the “gray zone,” making basal-only protocols viable in most cases [1].

#### 2.1.2. Calcitonin Thresholds and Clinical Decision Limits

Notably, men and women exhibit consistent differences in basal calcitonin levels, driven by biological factors that significantly inform both clinical interpretation and diagnostic thresholds. These differences are likely related to the different mass of C-cells in males compared to females (ratio ~2:1). Additionally, testosterone may stimulate C-cell proliferation. In contrast, no analogous effect is observed with female sex hormones [22]. An extensive population study (n ≈ 10,500 subjects) showed a mean calcitonin value of ~2.3 pg/mL in men vs. ~1.9 pg/mL in women (*p* < 0.001), even after excluding confounding conditions, with 95th percentile reference thresholds for healthy non-smoking adults of ~5.7 pg/mL in men and ~3.6 pg/mL in women, respectively. Accordingly, gender-specific **calcitonin** thresholds are required to avoid biochemical misclassifications [23]. Similarly, gender-specific peak thresholds should be generated for pentagastrin/calcium stimulation tests [i.e., ~80 pg/mL (women) vs. ~500 pg/mL (men)] [22]. Finally, smoking slightly elevates **calcitonin** in males (non-smoker ~2.4 pg/mL vs. smoker ~2.8 pg/mL; *p* < 0.001) while women show no smoking-related increase. Thus, in men reference thresholds may be further stratified by smoking status (e.g., smoker cutoff ~7.9 pg/mL vs. 2.8 pg/mL) [23]. All in all, applying higher male thresholds reduces unnecessary follow-ups and surgeries, while maintaining high detection rates for MTC and minimizing overdiagnosis. Accordingly, false-positive rates of ~1.8% in men vs. ~1.3% in women were reported in a large screening cohort [24]. Importantly, with new sensitive assays and sex-adjusted interpretation, many guidelines consider avoiding stimulation tests in truly basal-negative individuals (i.e., basal only protocols). Meta-analyses cite pooled basal calcitonin sensitivity/specificity ~99%, reinforcing the effectiveness of these improved tests [25].

#### 2.1.3. The Diagnostic Performance of Circulating Calcitonin

The sensitivity and specificity of basal calcitonin for detecting MTC range from 83 to 100% and 94 to 100%, respectively [26]. Meta-analyses support its reliability for thyroid nodule evaluation [25] and one Cochrane review reported high accuracy, though it noted heterogeneity and risk of bias [27]. Rarely, MTC may present with normal basal and even stimulated results in about 1–2% of cases [28]. Possible causes include impaired secretion, cryptic calcitonin isoforms undetected by immunoassays, or the analytical hook effect [26]. In such challenging cases, alternative biomarkers and confirmatory assays can unmask occult disease. This **calcitonin**-negative variant may respond well to standard surgical treatment, but rigorous long-term follow-up is essential as poorly differentiated cases may also occur with negative CT and are associated with worse outcomes [29].

#### 2.1.4. The Role of Serum Calcitonin in Screening and Diagnosis of Medullary Thyroid Carcinoma

Elevated basal CT (above gender- and assay-specific thresholds) indicates MTC with a nearly 100% positive predictive value. In the context of thyroid nodules, CT is a marker for early detection of occult MT that might elude US and/or cytology [25]. Notably, thyroid nodules are very common while MTC is a rare tumor and many non-MTC-related conditions may increase CT levels, leading to potential false-positive results. In addition, other conditions such as the hook effect can lower CT levels, leading to false negative results [25,30]. The European Thyroid Association recommends measuring calcitonin levels in patients scheduled for surgery or thermoablation of thyroid nodules, those with indeterminate cytology, or those with suspicious US findings. Cutoff points of 30 pg/mL in females and 34 pg/mL in males are also proposed to distinguish between non-MTC reasons for increased calcitonin and MTC. Such thresholds have been established for one assay platform and several additional variables may affect these results, making these thresholds nonactionable outside the authors’ setting [31]. The American Thyroid Association leaves clinicians to decide if and when to measure calcitonin in patients with thyroid nodules [1]. Overall, recommendations on calcitonin screening still differ between available guidelines, and the use of calcitonin screening of MTC varies significantly across different countries, specialties, and clinical centers. A broad consensus exists only on the pivotal role of calcitonin screening in cases with a family history of MTC or multiple endocrine neoplasia type 2, and its use in guiding the timing of prophylactic thyroidectomy in carriers of RET pathogenic variants [32,33].

#### 2.1.5. The Role of Serum Calcitonin in Postoperative Monitoring of Medullary Thyroid Carcinoma

After total thyroidectomy calcitonin levels typically decline within hours, and their normalization within weeks signals biochemical remission. Persistent or rising **calcitonin** postoperatively indicates residual or recurrent disease. Serial measurements allow calculation of doubling time (CDT), a powerful prognostic tool: CDT < 6 months correlates with poor survival, while CDT > 2 years indicates favorable outcomes [34]. Adjunctive biomarkers, especially CEA, may refine follow-up and prognostic stratification. Increased CEA levels and shortened doubling time are early signs of disease progression and the shift toward a more aggressive phenotype [35]. Accordingly, the follow-up of patients without an excellent response is based on the simultaneous measurement of both **calcitonin** and CEA, as well as their doubling time evaluation (Table 2) [1].

### 2.2. Procalcitonin

Procalcitonin (ProCT) is a 116-amino acid prohormone, normally cleaved to calcitonin in thyroid parafollicular C-cells. Under normal physiological conditions, ProCT expression is limited to thyroid C-cells and circulates at low concentrations. Unlike calcitonin, ProCT exhibits a stable and concentration-independent half-life of ~20–24 h in vivo [36] and it shows excellent in vitro stability when collected as serum or plasma samples, reducing pre-analytical variability [37]. Kratzsch and colleagues tested two fully automated and one non-automated calcitonin assays, comparing these results with those from ProCT (Brahms Kryptor) [38]. They evaluated pre-analytical factors and ProCT cross-reactivity in serum samples from 437 patients presenting with clinical conditions linked to elevated calcitonin levels. Their study also included 60 post-thyroidectomy patients and established calcitonin cutoff values for pentagastrin stimulation tests in 13 patients with chronic kidney disease and 10 patients with MTC.

Their findings revealed several key points. Serum calcitonin concentrations showed significant degradation when stored at room temperature for over 2 h or at 4 °C for more than 6 h. The cutoff values for both baseline and stimulated calcitonin varied depending on the specific disease state and assay methodology employed. Proton pump inhibitor treatment was the most common cause of elevated calcitonin levels. Notably, ProCT concentrations were elevated in MTC patients compared to those with chronic kidney disease who did not have concurrent infections.

The study concluded that elevated calcitonin concentrations occur frequently in patients without MTC and advocated for incorporating ProCT assessment when evaluating unexplained calcitonin elevations in ambiguous clinical scenarios. ProCT testing proves valuable when calcitonin measurements are compromised by pre-analytical or analytical limitations, or when calcitonin values fall within indeterminate ranges [39]. Moreover, in contrast with the weak agreement between different calcitonin assays [12,40] a high degree of agreement was demonstrated between ProCT assays, making it possible to establish general ProCT thresholds in a clinical setting. Lippi and colleagues showed that 10 fully automated commercial ProCT assays deliver acceptable correlations in 176 routine lithium heparin plasma samples, suggesting that other ProCT assays may also be appropriate for the purpose [41]. All in all, ProCT circumvents many of the analytical and pre-analytical problems associated with calcitonin measurement and has recently emerged as a potential complementary or alternative MTC biomarker.

#### Procalcitonin in Diagnosis and Monitoring of Medullary Thyroid Carcinoma

Multiple studies have robustly demonstrated that ProCT identifies MTC cases with high diagnostic accuracy. Results of a pivotal paper in the field [41] and the largest published series in either diagnostic [42,43] or postoperative settings [44,45] are summarized in Table 3, as well as those of the largest meta-analysis on the role of ProCT in MTC patients [46].

Algeciras-Schimnich and colleagues firstly demonstrated the potential role of ProCT as an alternative biomarker of MTC in a study including 835 patients with active MTC, cured MTC, neuroendocrine tumors, active and cured follicular cell-derived thyroid carcinoma, benign thyroid diseases, mastocytosis, and normal volunteers, respectively [42]. The Receiver Operating Characteristic (ROC) curve analysis revealed no significant difference in diagnostic performance between calcitonin and ProCT. A comparison of calcitonin and ProCT in stability studies showed that calcitonin was very unstable in vitro (i.e., a decrease of 35–50% from the original value 24 h after sampling). In contrast, ProCT concentrations did not significantly change during this time.

Giovanella and colleagues evaluated the role of routine measurement of ProCT in a sample of 1236 patients and reported serum calcitonin levels > 10 pg/mL in 14 (1.1%) patients [43]. Among them, two were found to have MTC, one a C-cell hyperplasia, while MTC was excluded in the remaining patients by subsequent clinical and histological follow-up. Basal levels of calcitonin were >100 pg/mL in two MTC patients, and pentagastrin-stimulated calcitonin was >100 pg/mL in two MTC patients and two non-MTC patients, respectively. Moreover, basal and stimulated ProCT values were elevated in only two MTC patients, resulting in 100% accuracy. As the main result, ProCT was detectable in MTC patients and undetectable in the remaining ones (100% sensitivity and 100% negative predictive values).

In a large prospective study Giovanella and colleagues evaluated 2705 patients with thyroid nodules, measuring only ProCT as a screening test. A ProCT cutoff of 0.155 pg/mL yielded 100% sensitivity and 99.7% specificity in diagnosing MTC [44]. Finally, Clausi and colleagues recently reported results obtained in 478 patients undergoing thyroidectomy [48]. Serum levels of ProCT and calcitonin were measured before surgery. Overall, 23/478 (4.8%) patients tested positive for MTC. Calcitonin values > 10 pg/mL demonstrated a sensitivity of 0.91, specificity of 0.98, PPV of 0.70, and NPV of 0.99. ProCT showed sensitivity, specificity, PPV, and NPV of 0.87, 0.96, 0.56, and 0.99 using a cutoff of 0.04 ng/mL, and 0.78, 0.98, 0.72, and 0.99 using a cutoff of 0.07 ng/mL, respectively. Interestingly, 80.9% of patients with a calcitonin value between 10 and 100 pg/mL would have been correctly identified as MTC or non-MTC by positive or negative ProCT using the cutoff 0.04 ng/mL. The authors concluded that calcitonin demonstrates superior sensitivity compared to ProCT as a diagnostic marker for MTC; however, implementing a two-step approach with ProCT as an adjunctive marker can enhance diagnostic precision and prevent overtreatment, especially when calcitonin serum concentrations fall between 10 and 100 pg/m. Such results are in line with those obtained by Giovanella and colleagues measuring calcitonin and ProCT in 16 patients with active (i.e., primary tumor before surgery or post-surgical recurrent disease) and 23 with inactive (i.e., complete remission) MTC, 125 patients with non-MTC benign thyroid disease, and 62 patients with non-MTC thyroid cancers [49]. Both calcitonin and ProCT median values were significantly higher in active (94 pmol/L and 1.17 ng/mL) than inactive (0.28 pmol/L ng/mL and 0.06 ng/mL), benign (0.37 pmol/L and 0.06 ng/mL), and malignant non-MTC diseases (0.28 pmol/L and 0.06 ng/mL), respectively. Notably, undetectable ProCT was found in five non-MTC patients with false-positive calcitonin results. The authors concluded that, at the very least, ProCT is useful in patients with positive calcitonin results, as negative ProCT values securely exclude active MTC (i.e., a rule-out test). As both markers are available on the same automated platforms, a reflex or reflective strategy can be easily adopted to refine the laboratory diagnosis.

Postoperative monitoring also benefits from ProCT measurements: among 55 patients under surveillance a 0.32 ng/mL threshold achieved 92% sensitivity and 98% specificity for residual or recurrent disease [45].

More recently, Censi and colleagues retrospectively evaluated 90 MTC patients during postoperative follow-up and found a strong relationship between calcitonin and ProCT, corroborating the relevant role of ProCT as an adjunctive biomarker to calcitonin, especially to exclude MTC structural recurrences in patients with a slight to moderate increase in calcitonin concentrations [46]. Importantly, the ProCT-to-calcitonin ratio has also demonstrated predictive value for progression-free survival, further validating its clinical utility as a prognostic indicator [50]. Collectively, available evidence indicates that ProCT measurement offers excellent diagnostic sensitivity, specificity, accuracy, and prognostic information, while providing significant pre-analytical and analytical advantages. Notably, a recent meta-analysis including 5817 individuals (335 with MTC) reported a pooled sensitivity of 90% and specificity of 100% for ProCT in MTC, providing robust evidence in favor of the use of ProCT in diagnosis and follow-up of MTC [47].

Indeed, ProCT levels can become markedly elevated during severe bacterial infections and sepsis due to ectopic production by non-thyroidal tissues. The Food and Drug Administration has approved ProCT assays for risk stratification in critically ill patients at risk for severe sepsis and septic shock [51]. Nonetheless, elevations due to bacterial infections necessitate careful interpretation, as in inflammatory states ProCT can markedly increase [52]. Table 4 summarizes the role of ProCT in different clinical settings and practical advantages.

Regarding the sensitivity of ProCT, which has been found inferior to that of calcitonin by some authors, it is worth noting that serum calcitonin measurement is integral (and often the only diagnostic biomarker) in establishing the diagnosis and referring patients to surgery. Obviously, the diagnostic sensitivity of calcitonin is biased toward 100% (i.e., a slight increase in calcitonin levels prompts diagnostic work-up while normal calcitonin, in the presence of detectable ProCT not measured at that time, would not have required further controls). Accordingly, the best performance an alternative marker can achieve is equal to that of calcitonin, and for statistical reasons, it cannot surpass the current calcitonin standard. Indeed, a potential reservation against replacing calcitonin monitoring as the gold standard for MTC may be related to the opposition/resistance of the medical community. Indeed, ProCT is FDA-approved as a marker for lower respiratory tract infection. Because MTC is rare and obtaining regulatory approval for new indications is costly and complex, manufacturers currently favor its off-label use in this setting. Therefore, ProCT may reasonably be considered as a complementary marker in patients with thyroid nodules and positive calcitonin results (as a rule-out test) and in MTC patients with unclear postoperative calcitonin trends.

### 2.3. Carcinoembryonic Antigen

Medullary thyroid carcinoma arises from parafollicular C-cells, which express and release CEA in approximately half of cases. CEA is a ~200 kDa onco-fetal glycoprotein involved in cell adhesion and part of the CEACAM immunoglobulin family. CEA is normally expressed during fetal gastrointestinal development, with minimal expression in healthy adults (<2–4 ng/mL) [53]. It is typically quantified via standardized immunometric assays, which are analytically robust, reproducible, and less affected by interferences and the hook effect than calcitonin. Notably, CEA measurement is affected by benign conditions (i.e., inflammation, smoking, liver dysfunction conditions) and other malignancies (colorectal or lung cancer), requiring cautious interpretation. In particular, postoperative CEA is widely adopted as a reliable tumor marker and prognostic factor for colon cancer [54]. Even if CEA is not a specific MTC biomarker, its measurement remains useful in assessing the extension and progression of the disease before and after thyroidectomy. Baseline CEA levels are low in early, thyroid-limited MTC, precluding its use in screening and early diagnosis of MTC. However, CEA remains a valuable adjunct marker to monitor patients after surgery and stratify the risk of recurrence and death in patients with advanced disease. Preoperative CEA values > 30 ng/mL indicate a larger size of the primary tumor and extra-thyroid tumor extension. Moreover, CEA values greater than 100 ng/mL are associated with lymph node involvement, distant metastases, and a poorer prognosis [55]. Accordingly, serum CEA should be systematically measured before surgery in patients with confirmed or suspected MTC to inform the extent of resection and, particularly, lymph node dissection(s) [56]. Following surgery, MTC patients require evaluation for residual disease presence, localization of metastases, and progressive disease identification. Postoperative restaging is essential to distinguish low-risk MTC patients from those at high risk of disease recurrence. Postoperative calcitonin and CEA levels should be systematically documented. Postoperative undetectable calcitonin levels correlate with favorable outcomes. In patients with basal serum calcitonin levels below 150 pg/mL after thyroidectomy, persistent or recurrent disease is typically limited to cervical lymph nodes. Conversely, postoperative serum calcitonin elevation above 150 pg/mL necessitates comprehensive imaging studies, including neck and chest CT, contrast-enhanced MRI, hepatic US, bone scintigraphy, bone MRI, and PET/CT. The integration of CEA measurement is useful in this context as post-thyroidectomy failure of CEA levels to normalize—or an increase in levels—suggests residual disease, offering an early warning for recurrence even when calcitonin remains low [1]. Notably, CEA demonstrates relatively stable kinetics compared to CT, displaying less diurnal fluctuation and thus serving as a robust indicator of tumor mass. Additionally, a rising CEA, even in the absence of a CT increase, suggests a more aggressive disease phenotype (i.e., dedifferentiated disease) and may prompt more comprehensive metastatic workups using advanced imaging modalities such as [^18^F]FDG PET/CT [57,58]. Among patients with advanced disease, structural disease growth rate can be estimated and monitored through sequential imaging studies using RECIST criteria to document tumor size increases over time, and by measuring serum calcitonin or CEA levels at multiple time points to determine tumor marker doubling time (DT) [1].

### 2.4. Pro-Gastrin-Releasing Peptide

As reported in previous sections of the present paper, calcitonin is the standard of care in the diagnosis and monitoring of MTC, while serum ProCT is a potential alternative or at least complementary test to calcitonin in unclear cases being free from analytical limitations and interferences that may lead to false-positive or negative calcitonin results. The pro-gastrin-releasing peptide (ProGRP) is a stable precursor of gastrin-releasing peptide (GRP), a neuropeptide involved in tumor growth and differentiation. ProGRP is principally employed as a tumor marker of small-cell lung cancer [59]. ProGRP is generally measured using fully automated, non-competitive IMAs, which provide high reproducibility and offer pre-analytical advantages. Furthermore, it is stable in serum, is not subject to the hook effect, and is measurably preserved even with delayed processing. To date no reference method or international standard has been identified in the literature regarding the analytical performance of IMAs, and comparisons between them are sparse [60]. More recently, ProGRP has emerged as a promising adjunct biomarker in MTC, with initial studies demonstrating diagnostic performance similar to that of established markers (Table 5).

Overall, ProGRP has only moderate sensitivity (~80%) and cannot be used in MTC screening and diagnosis, considering the sensitivity of ~100% robustly demonstrated by either calcitonin or ProCT. Considering the high specificity, it should be considered to rule out false-positive calcitonin results, but again, ProCT already demonstrated a quite absolute negative predictive value in patients with indeterminate calcitonin results. Interestingly, ProGRP correlates with metastatic burden, and ProGRP levels are significantly higher in patients with nodal or distant metastatic MTC than in those with limited disease. Additionally, ProGRP levels drop post-surgery, aligning with effective resection, and serial ProGRP measurements mirror imaging responses more closely than calcitonin or CEA in patients under tyrosine kinase inhibitor therapy (i.e., vandetanib), making it a potential early indicator of therapy response or resistance [62]. Evidence remains fragmented and insufficient to support or refute its implementation in clinical protocols as available studies were retrospective with small sample sizes. Generally speaking, serum ProGRP is likely of limited value in the diagnosis of MTC with some promising preliminary data suggesting its application in advanced disease, especially in therapy response assessment. All in all, ProGRP can currently be considered as a candidate MTC biomarker, and its inclusion in advanced MTC management protocols needs to be studied in a large prospective cohort of patients with progressive disease and active systemic treatment.

### 2.5. Carbohydrate Antigen 19.9

Carbohydrate antigen 19.9 (CA 19.9) is a sialyl Lewis-A glycan, classically used in monitoring pancreatic and gastrointestinal malignancies. Its aberrant expression was reported in subgroups of MTC characterized by aggressive or metastatic behavior [69]. CA 19.9 is measured by immunoassays employing monoclonal antibodies against the sialyl Lewis-A epitope. Inter-method variability persists even after standardization due to epitope heterogeneity, differences in proprietary antibodies, and technical variations. Additionally, approximately 5–10% of individuals lack the Lewis antigen and cannot produce CA 19-9, yielding potentially false-negative results [70]. CA 19.9 elevations may also be noted in benign hepatobiliary and pancreatic diseases and benign respiratory diseases, which may complicate interpretation in MTC patients [69]. Notably, CA 19.9 is not sensitive to early or localized MTC, as only 5–6% of intra-thyroid tumors overexpress it and fewer present with elevated CA 19.9 serum levels. Vice versa, serum CA 19.9 increases in advanced MTC and its serum concentration correlates with structural disease progression and total tumor volume. Immunohistochemical analyses confirmed CA 19.9 expression in metastatic MTC tissue, affirming the tumor origin of the circulating marker [71]. In a series of 122 MTC patients, mean CA 19.9 levels were significantly higher in those with progressive disease (median ~21.4 U/mL vs. ~7.3 U/mL; *p* = 0.01) and in those who died from MTC (*p* < 0.001) [69]. Additionally, prospective data identify CA 19.9 positivity and fast CA 19.9 DT (i.e., <6 months) in advanced MTC as independent predictors of mortality, regardless of imaging findings. In these patients, systemic treatment (e.g., with cabozantinib or vandetanib) may be considered sooner than imaging alone would indicate [72]. In conclusion, due to limited sensitivity and specificity CA 19.9 cannot replace established MTC biomarkers (i.e., calcitonin and CEA) but may serve as a useful adjunct in select CA 19.9-positive cases. Its elevation and rapid doubling convey increased mortality risk and may prompt earlier therapeutic intervention independent of imaging findings. Additional multicentric, prospective studies are needed to define optimal thresholds, assay standardization, and integration into MTC care algorithms.

## 3. Doubling Time of Calcitonin and Carcinoembryonic Antigen

Calcitonin and CEA DTs are used to assess tumor burden, the aggressiveness of MTC, and predict the likelihood of recurrence or progression. They help monitor the effectiveness of treatment and guide decisions about further treatment strategies. Patients with shorter DTs may be considered higher risk and may benefit from intensified surveillance and/or additional imaging and/or treatments. Calcitonin and CEA levels are assumed to increase exponentially in MTC, doubling over a specific timeframe. Reliable DT calculation requires a well-standardized procedure. First, serial tumor marker measurements (i.e., every 6 months) with a minimum of four values (ideally over 2 years) are recommended. Second, methodological consistency is essential: all measurements should utilize the same laboratory and assay platforms to ensure accuracy. Third, DT should be calculated using the following formula: DT = ln(2)/b, where “b” represents the exponential growth rate constant derived from regression analysis of measured Calcitonin and CEA levels. Fourth, values below the limit of quantitation (LoQ) of the assay are reported as “equal to the LoQ” (i.e., if the LoQ of a calcitonin assay is 2 pg/mL and the patients’ value is rendered as <2 pg/mL then the value inputted for calculation will be 2 pg/mL). Finally, undetectable values and detectable values that do not double are designated as “never doubled”. Multiple reliable platforms are freely available to calculate DTs. The Kuma Hospital DT Calculator (Kuma Hospital, Kobe, Japan) is widely employed and was also adopted by the authors of the present review [73]. A note of caution is necessary when using DTs in clinical practice as they can vary significantly between patients and other factors (i.e., tumor grade, stage, and site of metastasis). Accordingly, DT results should always be considered in the general clinical context, taking into account the results of other diagnostic tools. Interpretation criteria of calcitonin and CEA doubling times are summarized in Table 6.

Finally, absolute levels of calcitonin and CEA, as well as the ratio of DTs, are very useful in guiding advanced molecular imaging in patients with relapsing or advanced MTC. In general, [^18^F]DOPA PET/CT appears to be superior to [^18^F]FDG PET/CT in detecting and locating lesions in patients with recurrent MTC, especially when calcitonin exceeds 150 pg/mL, CEA is ≥5 ng/mL, or calcitonin DT is between 1 and 2 years. [^18^F]FDG PET/CT showed a better accuracy in patients with very high calcitonin levels (>500 pg/mL), those with a disproportionate CEA increase compared to calcitonin, and/or calcitonin/CEA DTs are <1 year. Patients with rapidly shortening calcitonin/CEA doubling times may also benefit from [^68^Ga]DOTA-peptides PET/CT for the detection of occult recurrence. Overall, calcitonin and CEA kinetics can serve as a trigger for advanced molecular imaging, improving the localization of small-volume or occult disease and better identifying high-risk patients who require more careful surveillance [74,75,76].

## 4. Measurement of MTC Biomarkers in Fine-Needle Aspiration Washouts

The measurement of **calcitonin** in fine-needle aspiration washout fluids (FNA-calcitonin) from thyroid nodules and/or lymph nodes suspected of MTC or lymph nodes has been proposed to circumvent limitations of cytology in detecting MTC with a sensitivity of only 55% to 65%. Despite numerous criticisms (i.e., interferences, lack of standards, analytical variability of different assays) affecting the accuracy of the results and making it hard to compare studies, all available studies show significantly high sensitivity and specificity of FNA-calcitonin which are clearly better than that of cytopathology (Table 7) [77].

Obviously, the appropriate sampling is key, and samples should be representative of the approached lesion in the thyroid nodules, thyroid bed, or lymph node, respectively [83]. Unlike FNA cytology, diagnosis remains possible using FNA-calcitonin even when no thyroid cells are aspirated since calcitonin exhibits high concentrations both within and surrounding the lesion area [77].

Finally, detectable levels of FNA-calcitonin (i.e., higher than the functional sensitivity of the employed assay) were found in most non-medullary thyroid nodules, likely due to normal C-cells entrapped in the FNA sample, especially from the middle/upper thyroid lobes, highlighting the relevance of an appropriate cutoff selection [83].

Relevant practical points for FNA-calcitonin measurement and interpretation are summarized in Table 8.

Recently, ProGRP measurement was tested in 212 patients with 235 thyroid nodules, classified into chronic thyroiditis, nodular goiter, papillary thyroid carcinoma, thyroid follicular neoplasm, follicular thyroid carcinoma, and MTC. Serum ProGRP and FNA-ProGRP were measured. The median serum ProGRP concentration was 124.40 pg/mL in MTC, significantly higher than in other tested groups. The serum ProGRP cutoff value was 68.30 pg/mL, yielding 53.85% sensitivity, 96.98% specificity, and a kappa value of 0.51 in MTC. The median concentration of FNA-ProGRP in MTC nodules was 2096.00 pg/mL, significantly higher than in other groups. ROC analysis of MTC nodules versus non-MTC nodules revealed a cutoff value of 22.77 pg/mL, achieving 94.12% sensitivity, 98.27% specificity, and a kappa value of 0.85. The authors suggested FNA-ProGRP measurement as an ancillary method for differential diagnosis between MTC and non-MTC thyroid nodules [63]. Further studies are required to confirm or refute this preliminary observation and accurately evaluate the diagnostic advantage over FNA-calcitonin. As a consequence, FNA-ProGRP measurement should only be considered in the context of clinical trials.

## 5. Integrated Use of Circulating Markers of Medullary Thyroid Carcinoma

This paper presents the biochemical and analytical characteristics of currently available MTC biomarkers, analyzing their diagnostic performance and clinical applications. Algorithms for the rational and integrated use of various biomarkers, as outlined in current guidelines, are presented below.

### 5.1. Screening and Diagnosis of MTC

**Figure 1:**
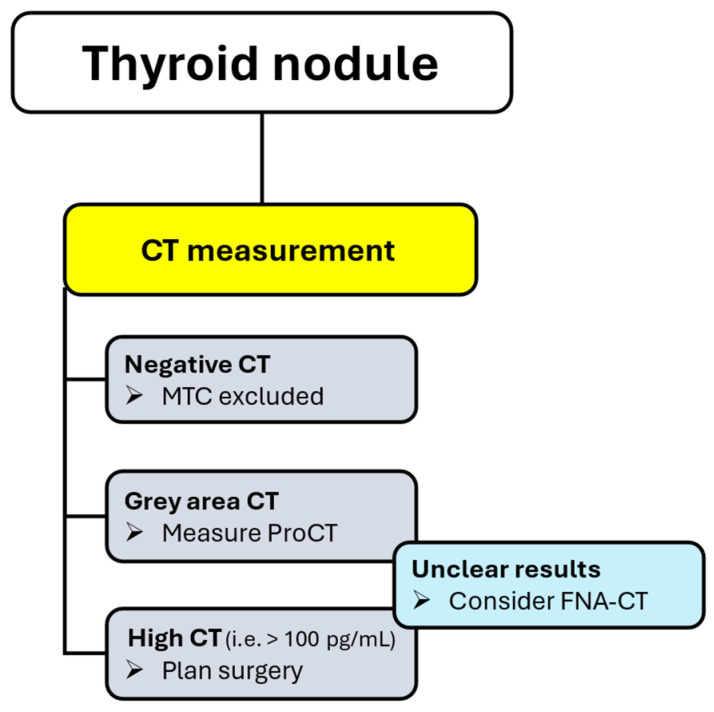
Screening and diagnosis of MTC in patients with thyroid nodules. **Legend**: MTC, medullary thyroid cancer; ProCT, procalcitonin; FNA-CT, fine-needle aspiration-calcitonin.

Calcitonin proved to be a useful screening test for the presence of MTC in patients with thyroid nodules, exhibiting a high diagnostic sensitivity. This practice remains debated due to the low prevalence of MTC and the non-negligible rate of false-positive calcitonin results [1]. Recently, ProCT has been shown to be highly accurate in excluding MTC in patients with indeterminate calcitonin values (i.e., 10–100 pg/mL), making a two-step procedure actionable. In turn, human, social, and economic costs associated with false-positive calcitonin results (i.e., imaging, FNA, additional laboratory tests) will be reduced by applying a reflex test procedure to safely rule out MTC in patients with undetectable ProCT (Figure 1) [47].

### 5.2. Preoperative Assessment

**Figure 2:**
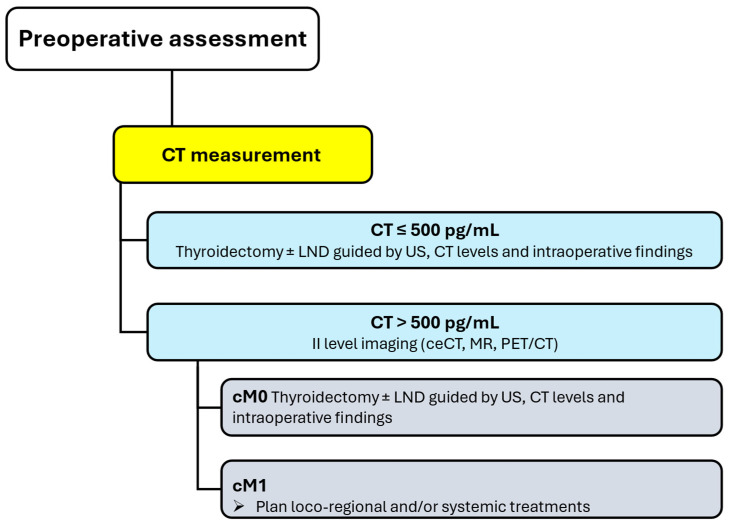
Preoperative assessment of MTC in patients. **Legend**: CT, calcitonin; LND, lymph node dissection; US, ultrasound; ceCT, contrast-enhanced computed tomography; MR, magnetic resonance; PET/CT, positron emission tomography/computed tomography.

Patients with diagnosed MTC should be assessed before surgery to map neck lymph nodes and detect/exclude distant metastasis. Distant metastases are unlikely in patients with negative US and calcitonin levels below 500 pg/mL. In such cases, no additional imaging is recommended, and total thyroidectomy should be planned with additional lymph node dissections informed by US, absolute calcitonin levels, and intraoperative findings. The risk of advanced disease and distant metastasis significantly increases in patients with calcitonin levels greater than 500 pg/mL, and level II imaging (i.e., ceCT, MR, PET/CT) is required to refine disease staging and detect or exclude distant metastases (Figure 2) [1].

### 5.3. Postoperative Monitoring

**Figure 3:**
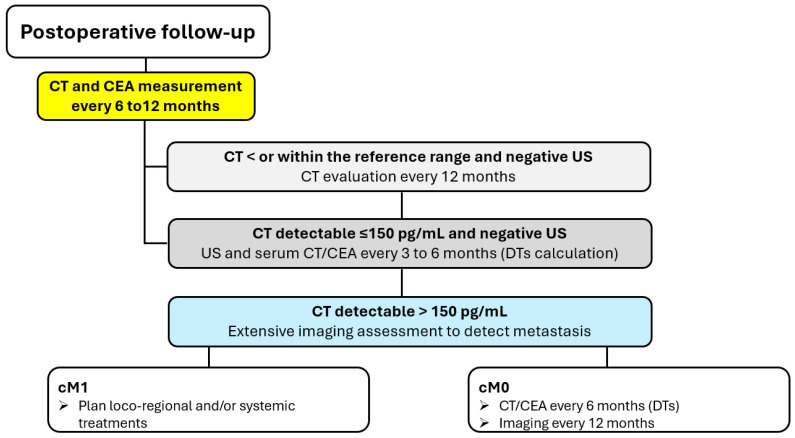
Postoperative assessment and follow-up of MTC patients. **Legend**: CT, calcitonin; CEA, carcinoembryonic antigen; US, ultrasound; DTs, doubling times.

Following surgery, serum calcitonin and CEA levels should be measured 3 months postoperatively, and if undetectable or within normal range, should be measured every 6 months for 1 year, and then annually. In patients with detectable serum levels of calcitonin and CEA following thyroidectomy, the levels of the markers should be measured at least every 6 months to determine their doubling time. Patients with elevated postoperative serum calcitonin levels of less than 150 pg/mL should undergo a physical examination and US of the neck. If these studies are negative, the patients should be followed up through physical examinations, measurement of serum levels of calcitonin and CEA, and US every 6 months. If the postoperative serum calcitonin level exceeds 150 pg/mL, patients should be examined by imaging procedures, including neck US, ceCT, MR, three-phase ceCT of the liver, and PET/CT (Figure 3) [1].

### 5.4. Analytical and Technical Challenges

Currently, fully automated immunoassays are widely used to evaluate MTC biomarkers across various analytical platforms, which are commercially available from different manufacturers. These highly automated systems offer an optimal balance of high throughput, rapid turnaround time, minimal sample volume, and cost-effectiveness [84]. Although significant advances have been made over the years in the analytical performance of various immunoassays, substantial inter-method variability still persists [85]. This variability necessitates the use of method-specific reference intervals, making it challenging to compare results obtained from different assays. Consequently, the use of the same immunoassay method is essential for reliable patient follow-up [84]. Although extensive research has been conducted over the years, challenges remain in terms of standardization and harmonization. Variability between methods can often be attributed to the use of primary antibodies with different epitope specificities. Indeed, significant discrepancies have also been observed between assays that utilize the same monoclonal antibodies, as seen in CA 19-9 assays employing the Centocor monoclonal antibody, indicating that other methodological factors also play a critical role [86]. In such cases, elements related to the assay’s architectural format must be considered when assessing inter-method variability. These include differences in incubation times, reaction kinetics, washing procedures, and the nature of the tracer system used. Together, these parameters influence the final numerical result, potentially leading to a lack of interchangeability between methods. To improve comparability and progress towards the harmonization and standardization of immunoassays for markers of MTC, efforts should focus on the development and implementation of reference materials and reference measurement procedures for each analyte. In addition to the development of methodological references, a key factor for harmonization between methods is the commutability of control materials, particularly those used in External Quality Assessment (EQA) schemes [85,87]. In order to improve this aspect, it is necessary to develop control materials prepared from authentic human samples or with advanced reconstitution technologies; check the commutability of EQA materials before distribution to laboratories (i.e., according to guidelines); use materials that are traceable to primary reference standards where available; and, overall, promote collaborations between reagent manufacturers, laboratories, and international laboratory medicine societies to create reference networks. Investment in this aspect would bring not only technical but also clinical benefits, improving the reliability of diagnostic results.

Table 9 summarizes the main aspects of the pre-analytical, analytical, and post-analytical phases concerning the tests used for determining circulating blood biomarkers of medullary thyroid carcinoma.

## 6. Discussion

Medullary thyroid carcinoma (MTC) is a neuroendocrine malignancy originating in the parafollicular C-cells of the thyroid and is characterized by the secretion of specific peptide markers. The biochemical profile of MTC serves as a cornerstone for diagnosis, risk stratification, and longitudinal monitoring. In addition to tumor markers such as calcitonin, ProCT, CEA and CA19.9, neuroendocrine MTC cells may also secrete different bioactive peptides and hormones that, in turn, can give rise to paraneoplastic syndromes (PNSs) [1]. Overall, PNSs are rare and mostly involve patients with extensive metastases and/or aggressive tumor biology. High levels of circulating calcitonin (>1000 pg/mL) are the most common biochemical cause of secretory diarrhea in MTC (prevalence ~5%), a debilitating condition with electrolyte imbalances (e.g., hypokalemia) and weight loss. Facial or generalized flushing (prevalence 2–5%) is associated with secretion of serotonin, prostaglandins, and CGRP, and mimics carcinoid syndrome. Elevated urinary 5-HIAA can support diagnosis. Ectopic ACTH syndrome (EAS) is a rare (<1% of MTC cases) but serious paraneoplastic phenomenon. It presents with rapid-onset hypertension, hypokalemia, proximal muscle weakness, and hyperglycemia. Diagnosis involves demonstration of inappropriately elevated plasma ACTH in front of increased cortisol levels, with lack of suppression on high-dose dexamethasone testing. Finally, MTC may also secrete histamine, contributing to flushing, and chromogranin A (CgA), a general marker of neuroendocrine activity. Elevated CgA has limited specificity and is not recommended for routine monitoring in MTC but may reflect tumor burden in select cases. Notably, paraneoplastic manifestations predict a worse outcomes, requiring intensive surveillance and early systemic intervention [1].

Regarding tumor markers “strictu senso”, which have been the central focus of our review, we can state that calcitonin remains the most specific and sensitive biomarker for MTC. Its serum concentration correlates well with tumor burden, lymph node involvement, and the presence of distant metastases. Moreover, postoperative calcitonin levels and doubling time are predictive of recurrence and survival, forming the basis for risk-adapted follow-up strategies. However, the utility of calcitonin is challenged in certain clinical scenarios and false-positive results may occur in various non-thyroid malignancies and non-malignant conditions or be induced by some pharmacologic agents. Furthermore, the lack of global assay standardization complicates inter-laboratory comparisons and undermines its longitudinal utility across different healthcare settings. In such a context ProCT has emerged as a promising biomarker with expanding utility beyond its traditional role in infectious diseases, and correlates significantly with MTC tumor burden, stage, and metastatic dissemination. Importantly, in cases where calcitonin levels are discordant with imaging findings or clinical progression, ProCT may provide additional insight. Its longer half-life and greater biochemical stability compared to calcitonin enhance its reproducibility and analytical robustness. Overall, the combination of calcitonin and ProCT measurements appears to improve diagnostic accuracy, particularly in patients with atypical biochemical profiles or aggressive disease variants. Despite its promise, ProCT’s utility is hindered by its potential for confounding elevations in the context of acute infection, systemic inflammation, or post-surgical stress. Nevertheless, these limitations can often be mitigated through clinical correlation and judicious interpretation. Carcinoembryonic antigen is frequently co-secreted with calcitonin and serves as a valuable adjunct in MTC, particularly for prognostic evaluation. Unlike calcitonin, CEA levels tend to rise in dedifferentiated or metastatic disease and may remain elevated in tumors that secrete little to no calcitonin. Short CEA doubling time (<2 years) has been associated with poor prognosis, making it a useful surveillance tool. However, its limited specificity due to non-MTC malignant and non-malignant causes of elevation precludes its use as a primary diagnostic marker. In contrast, markers such as pro-gastrin-releasing peptide (ProGRP) and carbohydrate antigen 19-9 (CA19-9) remain of limited clinical relevance in MTC but may have contextual significance in aggressive or dedifferentiated disease. ProGRP, a biomarker widely used in the diagnosis of small-cell lung carcinoma, has been sporadically investigated in MTC due to its neuroendocrine origins. Although some studies have suggested elevated ProGRP levels in advanced or poorly differentiated MTC, the evidence remains limited, and no standardized diagnostic thresholds or prognostic algorithms have been established. Similarly, CA19-9, a sialylated Lewis antigen primarily used in gastrointestinal and pancreatic malignancies, has shown rare and inconsistent elevation in MTC. Overall, these markers may be of interest in research settings or in patients with atypical tumor behavior but are not currently recommended for routine clinical use.

## 7. Integrated Approaches and Future Directions

The complex biology of MTC necessitates a multimodal monitoring strategy. The integration of calcitonin, ProCT, and CEA, particularly with attention to their respective doubling times, enables more nuanced disease tracking and risk stratification. Future efforts should focus on defining MTC-specific cutoffs and establishing standardized algorithms that incorporate biochemical, imaging, and molecular parameters. The integration of biochemical and molecular markers, inflammatory/immunologic signatures, and molecular imaging represents a promising step toward personalized risk stratification and tailored treatments in MTC. Ongoing clinical trials evaluating RET inhibitors and immunotherapy in MTC provide an opportunity to validate multi-marker panels that complement and extend beyond traditional biochemical biomarkers. Next-generation sequencing (NGS) has become an important tool in the molecular characterization of MTC. While RET proto-oncogene pathogenic variants are the defining genetic hallmark of hereditary MTC, NGS allows comprehensive analysis of both germline and somatic alterations, offering insights beyond conventional RET testing. It may also inform eligibility for selective RET inhibitors which show high response rates in RET-mutant MTC. Ongoing studies are also exploring the predictive value of co-mutational landscapes (e.g., TP53, TERT promoter pathogenic variants) for resistance to targeted therapies. Finally NGS of circulating tumor DNA (ctDNA) is emerging as a non-invasive tool to monitor minimal residual disease and assess clonal evolution under therapy (i.e., liquid biopsy). Integration of NGS data with immunogenomic profiling may further refine risk models and identify patients who could benefit from immunotherapy in combination with RET inhibition (i.e., VEGF, soluble immune-checkpoint molecules, markers of angiogenesis). Moreover biochemical and molecular marker panels are increasingly used in conjunction with functional and imaging methods to improve detection sensitivity and informs therapeutic timing. Until such technologies become widely accessible, optimizing the use of currently available markers remains essential to improving outcomes in this challenging thyroid malignancy.

## 8. Conclusions

We provided a robust analysis and a structured guide for serum biomarker deployment in MTC management, balancing established markers (i.e., calcitonin and CEA) and emerging tools to enhance clinical decision-making (i.e., ProCT, CA 19.9, ProGRP). The currently available panel of different markers is well-suited to manage MTC patients and guide treatments. Standardization of methods should be further ameliorated and large prospective multicentric validation is desirable to refine clinical thresholds of different markers, evaluate candidate biomarkers, and address rare clinical scenarios, such as calcitonin-negative MTC.

## Figures and Tables

**Table 1:** RET-related syndromes: pathogenic variants/phenotypes correlations.

RET Codon/Exon	Syndrome	Clinical Features	Behavior
634 (Exon 11)	MEN2A	MTC, Pheochromocytoma, Hyperparathyroidism	Aggressive behavior
609, 611, 618, 620 (Exon 10)	MEN2A, FMTC	MTC ± Pheochromocytoma	Moderate aggressiveness
768 (Exon 13), 804 (Exon 14), 891 (Exon 15)	FMTC	MTC (only)	Low–moderate aggressiveness
918 (Exon 16)	MEN2B	Early, aggressive, MTC mucosal neuromas, marfanoid habitus	Early onset, highly aggressive

**Legend**: RET, rearranged during transfection; MEN, multiple endocrine neoplasia; FMTC, familial medullary thyroid carcinoma; MTC, medullary thyroid carcinoma.

**Table 2:** Follow-up of MTC patients after primary surgery (i.e., thyroidectomy ± neck dissections).

Follow-Up	ER	BIR	BIR	SIR
Early	Negative US Negative calcitonin	Negative US calcitonin ≤ 150 pg/mL	Calcitonin > 150 pg/mL	-Positive imaging -Positive biopsy
Long-term	*FU at 6 months* -Negative: US/CT 1–2/yr -Positive: see BIR or SIR	*Visit at 6 months* US/CT 1/six months calcitonin DT -Negative: check 2/yr -Positive imaging (SIR) -Calcitonin > 150 pg/mL (BIR)	*Imaging* -CT/MR/PET/CT -Positive: (SIR) -Negative: Calcitonin, CEA, and calcitonin DT 2–4/yr	-Surgery -EBRT -Thermal ablations -Systemic therapies

**Legend.** US, ultrasound; CT, computed tomography; MR, magnetic resonance; PET/CT, positron emission tomography/computed tomography; BIR, biochemical incomplete response; SIR, structural incomplete response; DT, doubling time; CEA, carcinoembryonic antigen; EBRT, external beam radiation therapy.

**Table 3:** Evaluation of the diagnostic performance of ProCT in MTC patients [different clinical settings].

Reference	Study (No of Patients)	Patients Cohort	ProCT Cutoff	Sensitivity	Specificity
Algeciras 2009 [42]	Retrospective (835)	Mixed benign vs. active and inactive MTC	0.10 pg/mL	91%	96%
Giovanella 2013 [43]	Retrospective (1236)	Thyroid nodules	0.10 pg/mL	100%	100%
Giovanella 2018 [44]	Prospective (2705)	Thyroid nodules	0.155 pg/mL	100%	99.7%
Trimboli 2018 [45]	Retrospective (55)	Postoperative/follow-up	0.32 pg/mL	92%	98%
Censi 2023 [46]	Retrospective (90)	Postoperative/follow-up	0.12 pg/mL	100%	84%
Giovanella 2021 [47]	Meta-analysis (5817)	Diagnosis/follow-up	0.10 pg/mL	90%	100%

**Legend:** ProCT; procalcitonin; MTC; medullary thyroid cancer.

**Table 4:** Procalcitonin: clinical role and practical advantages in medullary thyroid cancer.

Screening/diagnosis	Most studies consistently showed similar sensitivity/specificity of calcitonin and ProCT, with better NPV of the latter. Other studies showed superior sensitivity of calcitonin with ProCT serving as a rule-out test in patients with calcitonin concentrations within the gray area (i.e., 10–100 pg/mL)
Postoperative follow-up	Strong ability to detect residual/relapsing disease
Prognosis/Prediction	The ProCT/calcitonin ratio correlates with outcomes (overall and disease-free survival)
Technical advantages	Greater assay stability and less susceptible to pre-analytical issues

**Legend:** ProCT, procalcitonin.

**Table 5:** Available studies on ProGRP in MTC patients (8 studies, 3886 patients).

Study	Patients	Sensitivity	Specificity	TP	FN	FP	TN
Han XD 2021 [61]	360	96.2	99.3	101	4	2	253
Giovanella 2021 [62]	254	75.9	97.9	51	16	4	183
Liang 2020 [63]	2446	53.8	96.7	114	98	73	2161
Parra-Robert 2017 [64]	38	88.9	76.9	20	2	4	12
Torsetnes 2014 [65]	190	80.0	90.0	48	12	13	117
Miao 2023 [66]	236	71.4	92.7	71	29	10	126
Martins Fernandes 2025 [67]	64	88.9	97.9	17	2	1	44
Schonebaum 2023 [68]	278	70.4	99.6	59	24	1	194

**Legenda**: TP, true-positive; FP, false-positive; FN, false-negative; TN, true-negative; ProGRP, pro-gastrin-releasing peptide. Sensitivity and specificity are expressed as %.

**Table 6:** Interpretation of calcitonin and CEA doubling times.

Doubling Time (Years)	Risk of Structural Recurrence	Prognosis
<1/2	Present	Very poor, short survival times
<1	High/Present	Poor
1–2	Intermediate	Intermediate
>2	Low	Favorable
Never doubling	Very low	Good

**Table 7:** Diagnostic accuracy of FNA-**calcitonin** reported in the main studies.

	Lesions (n)	Assay	Cutoff, ng/L	Sensitivity, %	Specificity, %
Boi, 2007 [78]	36	CLIA	36	100	100
Kudo, 2007 [79]	14	NR	67	100	NR
Diazzi, 2013 [80]	60	CLIA	17	100	88.8
Trimboli, 2014 [81]	90	CLIA	39.6	100	100
De Crea, 2014 [82]	62	CLIA	10.4	89	100

**Legend:** CLIA, chemiluminescence immunoassay; NR, not reported.

**Table 8:** Key points for standardization of FNA marker measurement in washout fluids.

**Sampling**	Representative of the lesion [lymph node or thyroid nodule]
**Washing solution**	0.9% saline solution [1 mL]
**Collection**	Rinse the needle ≥ 2 times, collect the amount of washout fluid and keep on ice
**Pre-treatment**	Mix and centrifuge the sample
**Measurement**	Consider interferences and perform dilution or batching to detect “hook effect” in case of undetectable FNA-**calcitonin**
**Interpretation**	Use an assay-specific cutoff adapted to the local population

**Legend**: FNA; fine-needle aspiration.

**Table 9:** Main laboratory characteristics of biomarkers used in medullary thyroid carcinoma.

Biomarker	Pre-Analytical Aspects		Analytical Aspects		Post-Analytical Aspects
	Healthy Subject	Sample	Methods	WHO IS	Reference Interval Clinical Decision Cutoff
CA 19-9	No peculiar preparation	No peculiar recommendations [88]	Immunoassay	Not available	Method-dependent [89,90]
CEA	No peculiar preparation Blood levels influenced by smoking	No peculiar recommendations [88]	Immunoassay	WHO 1st IS 73/601	Method-dependent [87,91]
Calcitonin	No peculiar preparation Blood levels influenced by age, sex, BMI, and smoking	Instability at RT, ice-bath storage after blood collection Centrifuge and analyze preferably within 30 min of sampling	Immunoassay	WHO 2nd IS 89/620	Method-dependent [92,93]
ProCT	No peculiar preparation	Greater stability at RT than CT	Immunoassay	Not available	0.1 ng/mL [44]
proGRP	No peculiar preparation Blood levels influenced by age, BMI, and smoking	No peculiar recommendations [88]	Immunoassay	Not available	Method- and matrix-dependent [94]

**Legend**: BMI, body mass index; Ca 19-9, carbohydrate antigen 19-9; CEA, carcinoembryonic antigen; IS, international standard; ProCT, procalcitonin; proGRP, pro-gastrin-releasing peptide; RT, room temperature; WHO, World Health Organization.

## Data Availability

Not applicable.
